# Preventing alcohol use with a universal school-based intervention: results from an effectiveness study

**DOI:** 10.1186/s12889-015-1704-7

**Published:** 2015-04-09

**Authors:** Henriette Kyrrestad Strøm, Frode Adolfsen, Bjørn Helge Handegård, Henrik Natvig, Martin Eisemann, Monica Martinussen, Roman Koposov

**Affiliations:** Regional Centre for Child and Youth Mental Health and Child Welfare, UiT, the Arctic University of Norway, Tromsø, Norway; Department of Psychology, University of Oslo, Oslo, Norway; Department of Psychology, UiT, the Arctic University of Norway, Tromsø, Norway

**Keywords:** Adolescents, Alcohol, Prevention, Effectiveness, Multilevel analysis

## Abstract

**Background:**

The effectiveness of the universal school-based alcohol prevention program “Unge & Rus” [Youth & Alcohol] was tested by an independent research group. The program aims to prevent alcohol use and to change adolescents’ alcohol-related attitudes. The main outcome measure was frequency of monthly alcohol use, favorable alcohol attitudes, perceived behavioral control (PBC), positive alcohol expectancy and alcohol-related knowledge.

**Methods:**

Junior high school students (*N* = 2,020) with a mean age of 13.5 years participated in this longitudinal pre, post and one-year follow-up study with a quasi-experimental design, involving an intervention group and a comparison group recruited from 41 junior high schools in Norway. Multilevel analysis was used to account for the repeated observations (level 1) nested within students (level 2) who in turn were clustered within school classes (level 3).

**Results:**

Results showed an increased level of alcohol-related knowledge in the intervention group (*p <* .005) as compared to the comparison group at one-year follow-up. However, no significant difference in change was found between the intervention group and the comparison group in frequency of monthly alcohol use, alcohol-related attitudes, PBC or alcohol expectancy at one-year follow-up.

**Conclusions:**

This study offers adequate data on the effectiveness of a school-based alcohol prevention program widely implemented in Norway. Under its current method of implementation, use of the program cannot be supported over the use of standard alcohol curriculum within schools.

## Background

A public health priority of the World Health Organization is to prevent harmful use of alcohol [1]. Alcohol drinking is the most prevalent and socially acceptable form of substance use among young people and adults. Likewise, alcohol is the most frequently used intoxicating substance in junior high school [2,3]. Early onset of alcohol use is associated with problematic substance use in later adolescence and serves as a predictor of alcohol dependence and other mental health problems [4,5].

Alcohol interventions are an important priority within school-based prevention, but the effectiveness of alcohol prevention programs has been modest [6]. Meta-analytic findings showed a significant mean treatment group difference in 18 high quality alcohol interactive programs with a small mean effect size of Hedges’ *g* = 0.14 [7]. However, a more recent review documented that six out of eleven alcohol-specific trials showed significant reductions in alcohol use [6].

One school-based interventions widely implemented in Norwegian junior high schools is “Unge & Rus” [Youth & Alcohol]. This program shares several core components with successful interventions like the European Drug Addiction Prevention (EU-DAP) program, “Unplugged” [8,9]. The EU-DAP study concluded that the program, “Unplugged”, can delay progression to frequent drinking and reduce occurrence of alcohol-related behavioral problems in European students [9]. Both “Unplugged” and “Unge & Rus” are based on a Social Influence Model in which the students are asked to participate and share normative beliefs [10-13]. They both target adolescents at junior high schools and have a peer-led component in the standard intervention curriculum. Both interventions have a family component that includes parents, and both interventions offer training to teachers in order to aid program implementation.

### The “Unge & Rus” [Youth & Alcohol] intervention

The intervention was developed by Wilhelmsen [14,15] in cooperation with Henriksen [16] and has been mandatory in several municipalities in Norway since 2006. The intervention is free of charge and is easily accessible online to parents, teachers and students. The program owner Norwegian Knowledge Center for Drugs (KoRus North) manages the website www.ungeogrus.no where the program materials and delivery instructions are available and free of charge.

The intervention, “Unge & Rus”, which is a combined version of the two programs, "Ungdom og alkohol” [Young and alcohol] [14] and “Foreldresamarbeidet” [Parents Working Together] [16], has never been evaluated before.

The preparatory program "Ungdom og alkohol” [Young and Alcohol] was only designed for students and aimed to: 1) postpone student’s alcohol debut; 2) reduce experimenting with alcohol; and 3) influence alcohol behavior by affecting causes of drinking. The evaluation study [14] of "Ungdom og alkohol” randomly assigned four schools to each of three conditions: highly role-specified (HRS), less role-specified (LRS), and control condition. The HRS condition had twice the number of peer leaders per class and a more detailed program prescription than the LRS condition. The HRS group had significantly larger reductions in the outcomes of alcohol use than the control group, whereas the difference between the LRS and the control group did not reach statistical significance [14]. The difference in alcohol use between the highly role-specified intervention condition and the control condition was small (Cohen’s *d* = 0.13).

The other program, “Foreldresamarbeidet” [Parents Working Together], was designed for parents. The program aimed to increase the collaboration between parent’s/guardian’s and the school, to increase parent’s/guardian’s authority in setting limits for their adolescents, and to increase parent’s/guardian’s competence to communicate with their adolescents about alcohol. No effectiveness study of the parent component has been conducted. The program was tested in two junior high schools in the community of Bodø, 1997–1998 [16]. The majority of parents assessed the program as useful and as having led to positive experiences. The report showed that the program created opportunities for parents to set common boundaries for alcohol use. Additionally, the program increased the frequency and improved the quality of conversations about alcohol between the parent/guardian and their adolescent.

The purpose of the intervention, “Unge & Rus”, is to facilitate cooperation between the school and the parents to allow students to: 1) develop knowledge about alcohol and the ability to think critically about its use; 2) strengthen attitudes against the use of alcohol; 3) reinforce the ability to say no to alcohol; and 4) delay the first use of alcohol.

The educational strategy of “Unge & Rus” is problem-based learning. Students are actively involved in the program by working on five different components. The first component includes a cultural and traditional theme addressing the consequences of alcohol abuse and alternatives to alcohol use, with a focus on developing awareness of the influence that friends, family, community, and society can have. The first component aims to share knowledge and attitudes related to alcohol use in different cultures, thus enabling young people to make their own choices and better manage negative influences. The purpose of the second component is to educate students about norms for alcohol use, thus aiming to correct misconceptions among students; e.g., that young people have a tendency to overestimate peer drinking and drug use [17,18]. The third component aims to increase students’ knowledge about alcohol, what it is and how it works. The intension is to increase knowledge about the physiological effects of alcohol on the body, and the content of alcohol in various products. Educational components can be valuable when integrated with other interactive activities. The fourth component of the intervention seeks to increase resistance skills and the ability to handle drinking pressure. The fifth component involves working with alcohol-related attitudes.

The program “Unge & Rus” is adapted for Norwegian junior high schools (age range 13–16 years) and is recommended for implementation at 8th grade with a timeframe of 20 school hours of 45 minutes in addition to two parent meetings. The intervention is carried out by teachers who receive an 8-hour course from the Norwegian Education Agency, with theoretical and practical training on how to deliver the program in a classroom setting. The program engages students to work on individual assignments, group projects and homework, using tasks that are directly connected to alcohol use. The students use the program website (www.ungeogrus.no) while working their way through the program components.

### The W8 [wait] project

The W8 [wait] was the name of the research project commissioned by the Norwegian Directorate of Health to evaluate the effectiveness of the school-based alcohol intervention, “Unge & Rus”, as it is implemented among all 8th grade students in Oslo, the capital of Norway. The name W8 [wait] was created as an acronym to separate the research project from the intervention itself (“Unge & Rus”).

The effectiveness of the intervention, “Unge & Rus”, was examined by comparing schools in Oslo to a group of schools receiving the standard Norwegian junior high school curriculum, in order to test whether there were significant group differences in the outcome measures over time. The Norwegian authorities have made schools responsible for introducing information on drugs and alcohol abuse as part of the standard curriculum in junior high schools [19].

This study tested whether the rate of change in frequency of monthly alcohol use, alcohol-related attitudes, alcohol expectancy, alcohol-related knowledge, and perceived behavior control, differed between the intervention and the comparison group. This paper will provide details on an independent effectiveness study of the intervention, “Unge & Rus”.

## Methods

### Participants and procedure

The effectiveness of “Unge & Rus” was tested using a longitudinal pre, post and one-year follow-up study with a quasi-experimental design, comparing an intervention group to a comparison group selected from 41 junior high schools in Norway. The study was conducted in Oslo and Akershus. Oslo implemented the program as a mandatory educational program in all of the 47 junior high schools, of which 24 schools accepted the invitation to participate in this study as the intervention group. The 23 schools that did not participate either did not provide a response to the invitation or refused based on reasons such as lack of time and resources. The comparison group (17 schools) was recruited from neighboring municipalities in Akershus, according to geographic vicinity and other socio-demographic characteristics as provided by Statistics Norway (www.ssb.no/english). The eligible sample consisted of 4,356 students, whereas 2,020 agreed to participate in the study.

The baseline sample consisted of 1,574 eighth-grade students with a mean age of 13.46 years (*SD* = 0.68), of which 50.6% were girls. A total of 24.0% had consumed at least one glass of alcohol, 81.5% lived with both of their parents, and 86.7% participated regularly in organized activities. The composition of perceived religious affiliation was: 67.6% Christian, 9.9% Islamic, and 3.3% other religions, with a further 19.2% reporting no religious affiliation.

Each participating school was responsible for distributing envelopes to all students including a study invitation with information sheets; one assigned to the parents and one assigned to the student. Parents had to sign the written consent and return it to the school in order for their son/daughter to take part in the study. Data was collected by anonymous self-report online questionnaires, filled out during class time. Participating students were rewarded after each test with minor school-related profile articles like pens, candy and post-it pads, in addition to participation in a lottery where ten students won a tablet computer at the one-year follow-up. Descriptive information on program implementation was collected from the teachers (*N* = 47) using an online questionnaire at T2.

The baseline assessment was conducted in January, 2011 (T1). The intervention took place during the spring semester of 2011. The first post-test was conducted in May, 2011 (T2), and the one-year follow-up test was carried out in May of 2012 (T3). The study was approved by the Regional Committee for Medical Research Ethics.

### Measures

#### Socio-demographic characteristics

Demographic variables included the adolescents’ age at baseline, gender, family structure (e.g., living with two parents, one parent or other relatives), religion (Christianity, Islam, Other), friends (number of friends), and organized leisure activities (yes or no).

#### Alcohol use

The two questions measuring adolescents’ Alcohol use were adopted from Aas and Klepp [20]. The first question was, “Have you ever consumed a glass of alcohol?” coded “No” (0) and “Yes” (1). The second question was, “How often have you consumed at least one glass of alcohol during the past three months?” The categorical responses were recoded to represent a drinking frequency per 30-days. The original response categories and recoded versions were as follows: “no times” (=0), “1-2 times last three months” (=0.4), “once a month” (=1), “2-3 times a month” (=2.5), “once a week” (=4.3), “2-3 times a week” (=10.7) and “4 - 7 times a week” (=23.6).

#### Alcohol attitudes

Alcohol attitudes measured to what degree they found it acceptable for students of the same age to drink alcohol in various situations. The Alcohol Attitudes scale comprised a mean of five questions where lower scores represented more conservative attitudes towards alcohol use [21]. A sample question was, “Do you find it acceptable for an 8th grader to drink a glass of alcohol without any adults present?” The response categories ranged from “No, totally wrong” (1) to “Yes, it’s ok” (7). The Cronbach’s alpha for the Alcohol attitudes scale was 86.

#### Perceived Behavioral Control (PBC)

Perceived Behavioral Control (PBC) was measured by four items asking students to estimate the degree of PBC on a 7-point scale measured by questions such as, “If someone is offering me a glass of wine or beer, I don’t know/I know how to refuse”. The response categories ranged from “I don’t know any ways to refuse” (1) to “I know several ways to refuse” (7). Higher scores indicate higher resistant self-efficacy scores. The Cronbach’s alpha for the PBC scale was 77.

#### Alcohol Expectancy Questionnaire (AEQ – A)

Alcohol expectancy was based on a short and modified Norwegian version of the Alcohol Expectancy Questionnaire for Adolescents (AEQ-A, the social scale) [13,22]. The five items asked students to indicate their positive alcohol expectancy on a 7-point scale with items such as, “Many alcoholic drinks taste good” and “Parties become more fun when alcoholic beverages are consumed there”. The response categories ranged from “strongly disagree” (1) to “strongly agree” (7). AEQ-A had a Cronbach’s alpha of 75.

#### Knowledge

Knowledge regarding alcohol was measured with three items, each allowing four response alternatives (only one correct option). These questions were: “What is the age limit for buying beer and wine in Norway?”, “What does blood-alcohol concentration measure?” and “What is the name of the kind of alcohol used in beer, wine and spirits?” The variable was coded as 1 for all answers right, and 0 for other answers (0, 1 or 2 correct answers).

#### Dosage measures

Teachers from both the intervention and the comparison groups were asked, “Have you participated in the program training for “Unge & Rus” during the last two years?” and “Have you visited the website www.ungeogrus.no?” Response categories were recoded to represent how many hours had been spent on the website with a range from “Less than one hour” (=0.5) to “More than five hours” (=6). Teachers in the intervention group were additionally asked: “How many hours did you spend on “Unge & Rus” in your class?” Response options were recoded to represent the number of hours spent ranging from “1-5 hours” (=3) to “More than 30 hours” (=35); “How did you organize the work with the intervention for your students?” Response options were categorized as “Integrated as school-lessons”; or “Separate project”; or “Other – please specify”; “How many weeks were spent on “Unge & Rus” in your class?” Response options were recoded to represent the number of days used from “Less than a week” (=3) to “More than three weeks” (=25); “Was the peer leader training implemented at your school?” Response was registered as “Yes” or “No”. Teachers in the comparison group were additionally asked, “Have you been working with any alcohol curriculums during the last two years in your class?” The three response options were: “No”, “Yes, with “Unge & Rus” and “Other efforts – please specify”.

### Statistical methods

Data were analyzed using the Statistical Package for Social Sciences (SPSS 21.0). The structures of these data were expected to be hierarchical, since students from the same class tend to be more similar to each other than students from other classes in addition to the dependencies within students due to multiple observations per student over time. Students could also have similar responses because of student-level characteristics, or because of their teachers and the way that their particular teacher implements the program relative to other teachers. The level of within class dependency was therefore examined.

To test whether the rate of change in the outcome measures differed between the intervention and comparison group, multilevel analysis and generalized multilevel analysis were used. Three-level models were implemented with repeated observations (level 1) nested within students (level 2), and students clustered within school classes (level 3) (equation 1) [23]. On level 1 the outcome was modeled as a linear function of time. With the treatment group variable as a predictor on level 3, the composite multilevel of change looks like this:

$$ {Y}_{ijk}=\left({\gamma}_{000} + {\gamma}_{001}\cdot {\mathrm{Group}}_k + {\gamma}_{100}\cdot {\mathrm{Time}}_{ijk} + {\gamma}_{101}\cdot {\mathrm{Group}}_k\cdot {\mathrm{Time}}_{ijk}\right) + \left({R}_{0jk} + {U}_{10k}\cdot {\mathrm{Time}}_{ijk} + {R}_{1jk}\cdot {\mathrm{Time}}_{ijk} + {U}_{00k}+{E}_{ijk}\right) $$

*Y*_*ijk*_ = Outcome at measurement *i* for individual *j* in school class *k*.

γ_000_, γ_001_, γ_000_, γ_101_ = Fixed effects

*R*_0*jk*_, *U*_00*k*_, *R*_1*jk*_*, U*_10*k*_ = Random intercepts and slopes on level 2 (individuals) and 3 (classes)

*E*_*ijk*_ = The residual for measurement *i* for individual number *j* in school class *k*

The overall effects were predicted with the time variable coded continuously. We also tested whether there were group differences on the post-measurement survey. This analysis was still based on a longitudinal model, however, time was treated as a categorical variable in this case [24]. By varying the reference time point in the analysis, predicted group differences on each occasion can be estimated. The multilevel analysis used full information maximum likelihood estimation, a method that does not require an equal number of observations for all participants, so respondents with missing observations can be included in the analysis [25]. All continuous outcomes were comprised of summary scores created by calculating the raw scores across all individual items within each scale.

## Results

### Participant flow

**Figure 1:**
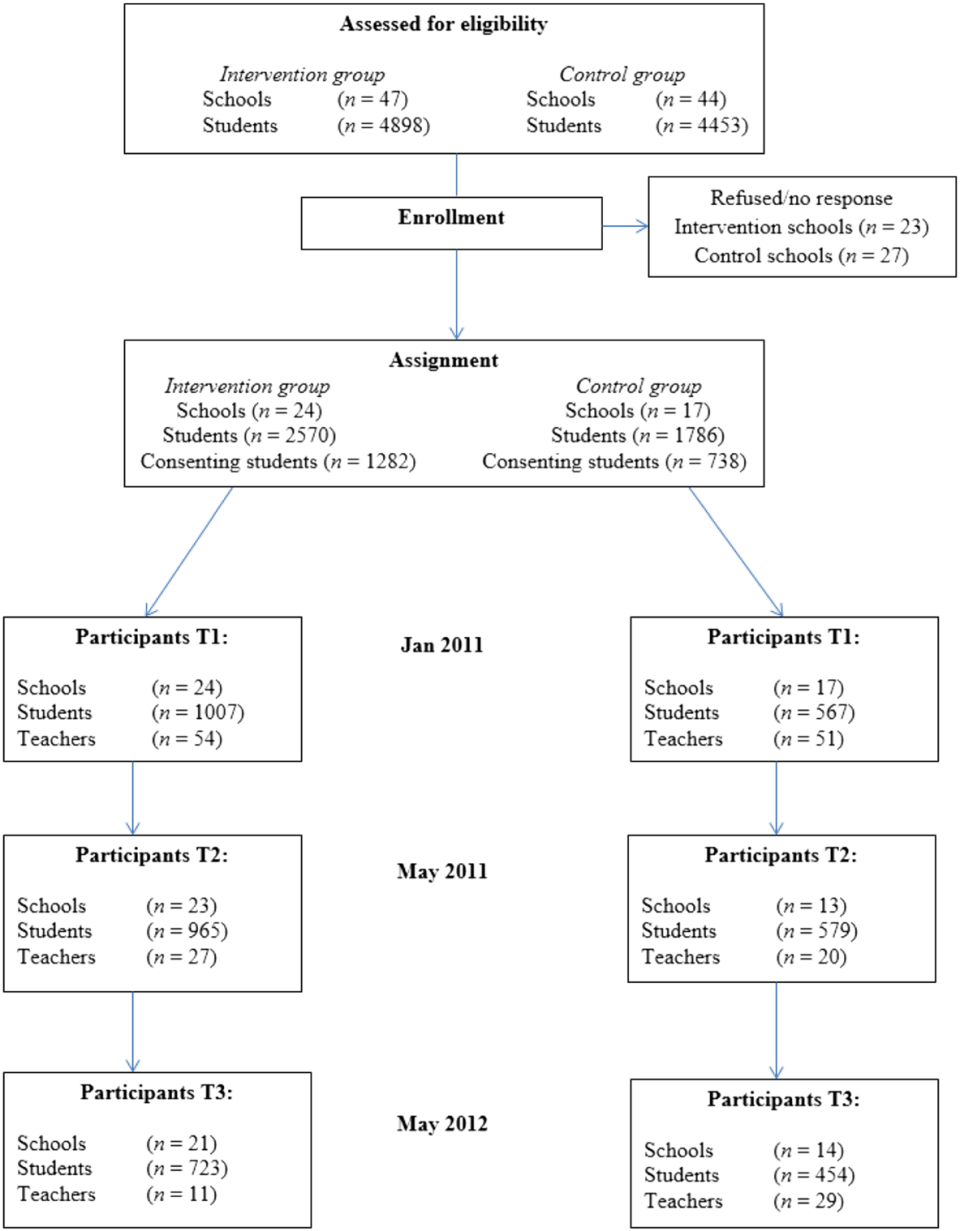
**Flow of participants.**

A total of 91 schools were eligible and invited to participate in the study. Fifty schools gave no response or did not participate due to the principal’s refusal explained by, e.g., lack of time and resources or participation in other programs and research within their school. Figure 1 illustrates the flow of participants through each stage of the study. A total of 41 schools finally accepted the study invitation, and invitations were sent to 4,356 students out of which 2,020 students agreed to participate. The sample of consenting students represents 21.6% of the total population of 8th grade students in the selected study area.

The response rate was calculated as the proportion of adolescents participating in each study assessment relative both to the the number of invited students and to the number of students who consented to participate. This resulted overall in a baseline response rate of 36.1% of invited students and 77.9% of consenting students (*n* = 1,574). Some students not participating at post-test may have participated in the follow-up. After the post-test and at the one-year follow-up, the response rates from participating students in the intervention group were 37.5% from invited students and 75.3% from consenting students at T2, and 28.1% from invited students and 56.4% from consenting students at T3, respectively. The response rates from students in the comparison group were 32.4% from invited students and 78.5% from consenting students at T2 and 25.4% from invited students and 61.5% from consenting students at T3.

### Attrition analysis

We compared participants who dropped out after the pretest (*n* = 190) with those who completed each measurement of the study (*n* = 750) on main outcome variables using multilevel analysis.

The amount of dropouts after the pretest did not differ between groups, with 9.3% from the intervention group and 9.8% from the comparison group. The amount of students who participated on each measurement time point in the study was higher in the intervention group (39.4%) than in the comparison group (33.5%). Results showed that students who dropped out after the pretest differed from those who completed in terms of more frequent monthly alcohol use (*t* = − 2.50, *p* = .01), with a small effect size (*d =* − 0.19), and lower scores in alcohol-related knowledge (*t =* − 2.95, *p* < .005), also with a small effect size (*d =* − 0.20). No significant differences were found for alcohol attitudes, PBC and alcohol expectancy. Results from the generalized multilevel analysis showed a difference between students who dropped out, as compared to those who completed, in higher onset of alcohol debut *OR* = 1.55 (*t* = 2.13, *p* < .05). Additionally, boys had 1.91 higher odds of dropping out than girls did (*t* = 3.88, *p* < .005). It is noteworthy that approximately 50% of individual dropouts at follow-up were explained by school class attrition, and equal numbers of schools were lost from baseline to one-year follow-up in both conditions. Overall, the attrition from consenting students was 22.1% at T1, 23.5% at T2 and 41.73% at T3, which is consistent with other longitudinal studies [26].

### Program dosage

Teachers from the intervention group reported that the program delivery was 17.9 (*SD* = 8.6) hours in class, and that they had spent 11.6 (*SD* = 6.5) days continuously on the program on average. About 2/3 (64%) of the schools had implemented the program as a separate project from other teaching in the classroom. A total of 92.6% (*n* = 25) of the teachers had also trained a peer leader within their class. The same amount of teachers reported that they arranged two parent meetings during the intervention period. The website (www.ungeogrus.no) was visited for 2.9 hours on average (*SD* = 1.9). During the last two years, 33.3% (*n* = 9) of the teachers in this study had participated in the Norwegian Education Agency program training.

The program website was visited by the comparison group teachers at an average of 0.8 hours (*SD* = 1.7). A total of 45% of the comparison-group teachers reported that no alcohol curriculum was delivered in class, while 45% had delivered a smoke-free campaign and 10% had delivered the “Unge & Rus” program during the last two years. None of the teachers from the comparison group had participated in the Norwegian Education Agency program training.

### Program impacts

The following presents descriptive results with means and standard deviations on the pretest, post-test and follow-up (Table 1), in addition to a summary of the multilevel analysis, showing the baseline and change statistics for overall effects measured from baseline to sixteen months (Table 2), and short-term effects measured from baseline to four months (Table 3). As the variable measuring “Knowledge” was coded as a categorical variable (dichotomous: correct on all items/not correct on all items), this variable was the only variable analyzed using generalized multilevel analysis (GLMM), and it was therefore not included in the tables presenting the other outcomes that were analyzed by multilevel analysis. As expected, the greatest degree of variance was at level two (within students, ranging from 12.8-54.4%). There was still a significant ICC at level three (within schools, ranging from 0.7-8.4%). These findings indicate that the three-level approach is appropriate.

**Table 1:** **Descriptive results**

**Measures**	**Pretest**		**Posttest**		**Follow-up**	
	**Intervention**	**Control**	**Intervention**	**Control**	**Intervention**	**Control**
	***M*** **(** ***SD*** **)**	***M*** **(** ***SD*** **)**	***M*** **(** ***SD*** **)**	***M*** **(** ***SD*** **)**	***M*** **(** ***SD*** **)**	***M*** **(** ***SD*** **)**
Alcohol use	4.14 (8.45)	4.85 (8.92)	4.64 (8.73)	5.62 (9.32)	5.40 (8.49)	6.10 (8.94)
	*n* = 999	*n* = 566	*n* = 960	*n* = 577	*n* = 720	*n* = 453
Alcohol	2.18 (1.33)	2.29 (1.38)	2.40 (1.50)	2.62 (1.58)	3.06 (1.88)	3.30 (1.78)
Attitudes	*n* = 987	*n* = 561	*n* = 963	*n* = 579	*n* = 723	*n* = 454
PBC	5.91 (1.30)	5.92 (1.25)	5.65 (1.50)	5.77 (1.36)	5.54 (1.63)	5.77 (1.22)
	*n* = 983	*n* = 558	*n* = 963	*n* = 579	*n* = 723	*n* = 454
AEQ-A	2.41 (1.25)	2.54 (1.29)	2.51 (1.43)	2.82 (1.54)	3.01 (1.68)	3.23 (1.50)
	*n* = 980	*n* = 556	*n* = 963	*n* = 579	*n* = 723	*n* = 454

*Note:* Alcohol use per 30-day frequency (0–23.6), Alcohol Attitudes (1–7), PBC (1–7), AEQ-A (1–7).

**Table 2:** **Multilevel model results for overall effects**

	**Alcohol use**	**Alcohol Attitudes**	**PBC**	**AEQ-A**
**Fixed parameters**				
*Intercept*	0.17 (0.07)*	2.17 (0.07)*	2.07 (0.05)*	2.30 (0.06)*
Group	−0.06 (0.09)	0.08 (0.10)	−0.04 (0.07)	0.19 (0.08)*
Time	0.02 (0.00)*	0.06 (0.00)*	0.01 (0.00)*	0.05 (0.00)*
Group x Time	−0.00 (0.01)	0.00 (0.00)	−0.00 (0.00)	−0.00 (0.00)
**Random parameters**				
*Level 1* Within subjects	3.84 (0.10)*	0.78 (0.03)*	1.07 (0.03)*	0.85 (0.03)*
*Level 2* Between subjects	0.57 (0.09)*	1.12 (0.06)*	0.55 (0.04)*	0.84 (0.05)*
*Level 3* Between classes	0.03 (0.03)	0.16 (0.04)*	0.03 (0.01)	0.10 (0.03)*

Note. *p < .05. Parameter estimates and standard errors (in parentheses). Intervention group = 1, Control group = 0. Time coded monthly: 0, 4, 16 months.

**Table 3:** **Multilevel model results for short-term effects**

	**Alcohol use**	**Alcohol Attitudes**	**PBC**	**AEQ-A**
**Fixed parameters**				
*Intercept*	0.45 (0.07)*	2.49 (0.07)*	2.24 (0.05)*	2.59 (0.06)*
Group	−0.08 (0.12)	0.17 (0.11)	−0.07 (0.08)	0.32 (0.09)
Time	0.21 (0.09)*	−0.26 (0.05)*	−0.14 (0.05)*	−0.16 (0.05)*
Group x Time	−0.00 (0.14)	−0.14 (0.08)	−0.07 (0.08)	−0.22 (0.08)*
**Random parameters**				
*Level 1* Within subjects	4.19 (0.11)*	1.05 (0.03)*	1.09 (0.03)*	0.99 (0.03)*
*Level 2* Between subjects	0.86 (0.09)*	1.18 (0.06)*	0.55 (0.04)*	0.88 (0.05)*
*Level 3* Between classes	0.04 (0.03)	0.19 (0.04)*	0.03 (0.01)*	0.11 (0.03)*

Note. *p < .05. Parameter estimates and standard errors (in parentheses). Intervention group = 1, Control group = 0. Time measured after four months.

#### Alcohol use

The intra-class correlation (ICC) in level 2 showed that 12.8% of the variance was between students of the same class. At level 3, the ICC showed that 0.7% of the variance occurred across classes. Baseline rates on frequency of alcohol use showed no significant difference between groups (*t* = − 0.69, *p* = .49). Short-term effects, measured four months after baseline, showed that the interaction term between group and time was close to zero and non-significant (*t* = 0.02, *p* = .99). The overall effects showed no significant time-by-group interaction on alcohol use (*t* = − 0.83, *p* = .41), indicating no evidence of a different development in the intervention group as opposed to the comparison group.

#### Alcohol attitudes

The ICC at level 2 showed that 54.4% of the variance was between students and, in level 3, the ICC showed that 8.4% of the variance occurred across classes. Students’ attitudes to alcohol showed no significant baseline difference between groups (*t* = 0.85, *p* = .39). Short-term effects showed that the interaction term between group and time was not significant (*t* = − 1.73, *p* = .08). The overall effect measuring attitudes toward alcohol use revealed that the interaction term between group and time was close to zero and non-significant (*t* = 0.61, *p* = .54), indicating that the groups did not develop differently.

#### Perceived Behavioral Control (PBC)

The ICC at level 2 showed that 33.3% of the variance was between students of the same class and, in level 3, the ICC showed that 1.9% of the variance occurred across classes. There were no significant baseline differences between groups (*t* = − 0.58, *p* = .56). The short-term effect measured after four months showed that the interaction term between group and time was close to zero and non-significant (*t* = 0.82, *p* = .41). The results also did not show an overall significant group-by-time interaction in PBC, when measured after sixteen months (*t* = − 0.21, *p* = .83). This implies that the two groups did not develop differently.

#### Alcohol Expectancy Questionnaire (AEQ – A)

The ICC at level 2 showed that 46.9% of the variance was between students and, in level 3, the ICC showed that 5.9% of the variance occurred across classes. Students from the comparison group had significantly higher alcohol expectancy at baseline (*t* = 2.20, *p* = .03). There was a significant group-by-time interaction in AEQ-A (*t* = − 2.71, *p* = .007) when measured after four months, showing that students from the comparison group developed more positive expectancies toward alcohol than students from the intervention group. Measured after sixteen months, the overall results did not show a significant group–by-time interaction in alcohol expectancy (*t* = − 0.05, *p* = .96).

#### Knowledge

The frequency of all answers correct among students in the intervention group and the comparison group was 33.8% and 39.4% at pretest, 40.8% and 40.3% at posttest, and 53.1% and 48.5% at one-year follow-up, respectively. There was no significant difference between the groups at baseline. The GLMM analysis measuring knowledge did not show a significant difference in the rate of change between the intervention and the comparison group in terms of number of students with correct answers (*t* = − 1.43, *p* = .153), measured after four months. A significant difference in the rate of change in alcohol-related Knowledge between groups measured from baseline to sixteen months (*t* = − 2.91, *p* = .004) was detected.

## Discussion

The aim of the current study was to evaluate the effectiveness of a universal school-based alcohol prevention program among junior high students in Oslo. The analysis examined the impact of the intervention on students from baseline to the one-year follow-up, a period of sixteen months.

Results showed that the development from baseline to one-year follow-up was not significantly different between the intervention and the comparison group, except for alcohol-related knowledge. The number of students with all knowledge items correctly answered increased more from baseline to one-year follow-up in the intervention group than in the comparison group. The test of short-term effects measured after four months showed no significant difference between groups, except in alcohol expectancy. Baseline rates were equal in both groups for all outcomes, apart from alcohol expectancy. The comparison group had higher alcohol expectancies at baseline and after four months, when compared to the intervention group. This finding indicates that the intervention may affect adolescents’ alcohol expectancies in the short term, whereas the effect does not last in the long term.

The absence of an enduring overall effect on alcohol expectancies may be due to several reasons, such as individual changes in beliefs about alcohol use as a socially acceptable behavior or the ability of an intervention to maintain its effectiveness one year after implementation. These findings are in accordance with previous research showing that alcohol-related knowledge can be increased and alcohol expectancies may be changed in the short term, while influencing drinking behavior in the long term is a difficult task [4,27-31].

This study showed an overall lack of effectiveness for the intervention, according to the program’s defined objectives. However, a longitudinal study among adolescents is expected to show that people in this stage of life have increased interest in alcohol use. The frequency of alcohol use in this study was low in both groups at baseline and, likewise, at the one-year follow-up. The relatively low frequency of alcohol use among the participants in this study can be explained by possible selection bias. At the same time, our findings are consistent with the European School Survey Project on Alcohol and Other Drugs (ESPAD), which reports Norwegian adolescents as the group with the lowest alcohol use among all 15 and 16-years-olds in Europe [32]. However, nearly 20 percent of the adolescents in this study had already experienced a debut of alcohol use despite their young age, and the large standard deviations in our results reveal a subgroup of more frequent drinkers. The level of within-school class dependency for alcohol use in this study was low. The majority of students in this study do not drink alcohol, so this explains the low variation between classes. The subgroup of frequent drinkers may well still drink with some of their fellow classmates (we can’t say from this study whether they do or not), but whether or not they do is on average not influenced by classroom-level explanatory variables. Adolescents are vulnerable to peer pressure, and research shows that alcohol use is predicted by having peers who consume alcohol [33,34]. The resistance to peer pressure, measured by PBC in this study, showed that the level of within-class dependency was relatively low. This could indicate that a school-based program does not necessarily influence the PBC among adolescents, but the level of PBC are more influenced by friends and family and not related to school experiences. On the other hand, this study found higher levels of dependency within classes in alcohol expectancies and alcohol-related attitudes. This could indicate that schools can have an impact on adolescents’ alcohol expectancies, and that they are in a position to promote preferable attitudes. However, this could also indicate a different implementation quality between schools. The previous version of this program demonstrated the effect of implementation quality showing a significant reduction in alcohol use when comparing a highly role-specified condition with the control condition, but did not find a significant difference between the low role-specified condition and the control condition [14].

Teachers’ descriptive reports on time spent using the program are in line with program procedures, but the relatively low attendance in program training might have contributed to a lower implementation quality in the schools. All participating schools are public schools with regular alcohol and drug education within their standard curriculums. The intervention group in this study received the “Unge & Rus” intervention in addition to the standard curriculum of drug prevention provided in Norwegian schools. The standard curriculum assumes that e.g., curriculums with natural sciences should provide information about risk factors related to drug use, and curriculums related to the social sciences should discuss consequences of drug use, and invite the students to reflect on different attitudes towards drugs. The comprehensiveness of these standard curriculums varies, as does the experience of each school implementing them. Health promotion and prevention through societal laws and regulations might influence both the parents and the adolescents to maintain a more restrictive approach to alcohol use [35]. Our findings revealed a generally low frequency of alcohol use among the sample. When the frequency of alcohol use is low in both groups, we cannot expect the intervention to have a high impact in relation to drinking behavior. However, these findings also revealed that there is a group of more frequent drinkers that may not be influenced by this type of intervention. Characteristics of those who drink more frequently were found in previous studies to be different from those who do not drink and include such factors as gender, smoking and religion [36-38].

Participants were recruited for the comparison group from schools in a neighboring municipality with a similar demographic pattern to those of the intervention group. The municipalities compared in this study, Akershus and Oslo, are often described as one region due to the similarities in their populations [39]. The participating schools from Akershus are located, to a larger degree, in rural areas than those from Oslo, and both municipalities are economically prosperous.

Even though the “Unge & Rus” program contains several key components that identify successful interventions [40], this study could not find a difference between students receiving the intervention as compared to students in the comparison group. Universal school-based prevention programs have, in general, been criticized for poor outcomes and low effect sizes [6,7,41,42]. Nonetheless, several evidenced-based alcohol prevention programs targeted towards adolescents do exist [43]. The majority of these programs use interactive designs that actively involve students [7], include structured activities and a parent component, and offer teachers training [34] in addition to on-line delivery [44], similar to “Unge & Rus”.

### Strengths and limitations

Some schools did not respond to the invitation and some principals refused based on reasons such as prioritization of time, resources, other programs or research within their school. There is also no guarantee that there are less socially acceptable reasons for not participating than those reported by the school principals. Such reasons are most likely unrelated to adolescent alcohol consumption and probably occur with similar frequency in both groups, which indicates that the results may be generalized, and that this type of missing data was unsystematic and equal across groups. We could not find any evidence of differential attrition between conditions. Students were tracked over time by a unique id-code and all participants were included in the analysis, regardless of their individual exposure to program activities in the intervention group. Dropout rates did not differ between the assigned conditions, but attrition was related to alcohol use at baseline. Losing more of the frequent drinkers to follow-up could therefore have had an impact on effect size measurements. Subject attrition in prevention research has generally shown that subjects who typically disappear from the study are more likely to be users than those who remain [45,46].

The current study has some limitations that should be pointed out. First, we evaluated the effectiveness of “Unge & Rus” in Oslo, where the program has been mandatory in schools since 2006. Schools from neighboring municipalities were invited to participate as part of a comparison group. Among teachers in the comparison group, 10% reported that they had previously received the program training. However, 10% of the teachers constitutes no more than one single teacher so it is unlikely that this could have influenced the results. Since the intervention schools were already selected, a randomized, controlled trial could not be conducted. Most of the evidence on alcohol research comes from quasi-experimental studies where the possibility of bias and confounding variable always exists, thus resulting in lowered internal validity [28].

Secondly, attrition in approximately 50% of the cases was explained by school class attrition. When school class attrition has occurred it’s more likely that the data collection has failed as a consequence of the teacher’s organization rather than student characteristics. Teachers were responsible to making it possible for the students to answer during school-time as they provided the link to the web-based questionnaire and the id-codes to each student, so if they missed one data collection the consequence could be attrition of a whole school-class. There could be other reasons, but it is less likely that this type of attrition correlates with the study outcome measures.

Thirdly, there was also a lack of adequate information about the quality of implementation. Implementation of a school-based program is not without challenges, so we cannot know for certain whether the program was delivered in a less-than-optimal manner, whether the program simply does not work in its present form, or whether the comparison group’s curriculum is perhaps equally as effective as the one evaluated in this study. If there is an implementation problem, it would be natural to perform follow-up studies on the dimension that emerged as critically important in the study by Wilhelmsen et al. [14]; namely, the degree of structure in the program implementation. The program activates students to work in groups, and a peer leader is in charge during those group activities. A peer-led activity might be less structured when compared to an activity led by a trained teacher who may influence the effectiveness of the program.

Fourthly, this study relied on self-report measures from a young sample. We cannot be sure that all adolescents fully understood the meaning of key words used in the questionnaire (e.g., alcohol and drunkenness). This might affect the construct validity of the questionnaire. However, in terms of reliability, studies of self-reported alcohol use suggest that these are reliable indicators of drinking behavior as indicated by high test-retest reliability [47,48].

Fifth, the variable measuring alcohol-related knowledge was not optimal. It turned out that there was little variation in the responses. In addition to a poor validity of the variable, it should have included more questions measuring knowledge of the adverse effects of alcohol.

## Conclusion

This study provides new information on the effectiveness of the “Unge & Rus” prevention program implemented in junior high schools in Oslo. The rate of change did not differ between participants receiving the intervention and those receiving the standard alcohol curriculum. Furthermore, as the program needs to be delivered in a regular school setting, these findings cannot support the use of the intervention, as it is currently implemented, over the use of standard alcohol curriculum.

The implications from our findings on prevention practice raise an important question as to whether the lack of results depends on the implementation process or the program content. Early onset of alcohol drinking predicts several risk factors for problem behaviors. Therefore, research on preventing alcohol drinking still needs to be improved. Decisions made by politicians and school administrators on implementation of evidence-based preventive interventions are, therefore, an important issue. In combination with health promotion, a school-based intervention has the opportunity to reach several contextual and cultural areas. Implementation of more specific interventions targeted towards selected groups and families could be more effective than the use of universal preventive school-based programs.
